# Digital Health Interventions for Sexual Health Education Among Adolescents With Autism Spectrum Disorder: Scoping Review

**DOI:** 10.2196/79009

**Published:** 2026-03-19

**Authors:** Elsi Rahmadani, Idris Adewale Ahmed

**Affiliations:** 1 School of Nursing & Applied Science, Lincoln University College, Petailing Jaya, Malaysia; 2 Department of Pediatric Nursing, STIKes Tri Mandiri Sakti Bengkulu, Jl. Raya Hibrida No.3, Sido Mulyo, Gading Cempaka, Bengkulu, 38229, Indonesia, 62 89504079722

**Keywords:** autism spectrum disorder, digital health, sexual education, adolescents, scoping review, PRISMA

## Abstract

**Background:**

Adolescents with autism spectrum disorder (ASD) experience persistent barriers to accessing comprehensive and developmentally appropriate sexual health education. Conventional curricula often fail to reflect their cognitive, social, and communication needs, increasing vulnerability to misinformation and sexual exploitation. Digital health interventions offer a promising avenue to deliver tailored, interactive, and accessible learning experiences for adolescents with ASD.

**Objective:**

This scoping review aimed to map and synthesize the evidence on digital health interventions designed to provide sexual health education to adolescents with ASD.

**Methods:**

A scoping review was conducted using Arksey and O’Malley’s framework, refined with Joanna Briggs Institute guidance and reported following the PRISMA-ScR (Preferred Reporting Items for Systematic Reviews and Meta-Analyses extension for Scoping Reviews) standards. In total, 6 databases (ie, PubMed, Scopus, CINAHL, ERIC, PsycINFO, and Web of Science) were searched from 2010 to June 2025. Eligible studies involved adolescents aged 10 to 19 years with ASD, used digital platforms to deliver sexual or reproductive health education, and were published in English. Two reviewers independently screened, extracted, and synthesized data using descriptive and thematic approaches.

**Results:**

A total of 16 studies met the inclusion criteria. Most studies were conducted in high-income countries and delivered content through video-based, web-based, or mobile modalities. Key features associated with positive learning outcomes included personalization, strong visual interactivity, and caregiver involvement. Reported improvements focused on sexual knowledge, behavioral understanding, and user acceptability. However, methodological limitations were common, including small and nonrepresentative samples, a lack of standardized outcome measures, and minimal gender-specific or culturally adapted content. Notably, co-design with autistic adolescents and implementation in low- and middle-income countries was scarce.

**Conclusions:**

Digital health interventions demonstrate promising early effectiveness for delivering inclusive, developmentally appropriate sexual health education to adolescents with ASD. To advance this field, future research must strengthen methodological rigor, include diverse and gender-balanced populations, use participatory design, and ensure cultural adaptability to support equitable access globally.

## Introduction

Adolescents diagnosed with autism spectrum disorder (ASD) often miss out on crucial sexual health education, despite ASD affecting more than 1% of young people worldwide [1]. The World Health Organization recognizes sexual health as a fundamental human right critical to psychosocial well-being [2]. Yet, adolescents with ASD commonly receive insufficient, delayed, or entirely absent sexuality education [3] during a developmental period marked by puberty, identity formation, and increasing social complexity.

Growing evidence shows that young people with ASD face distinct and systemic inequalities in sexual health education. These inequalities are characterized by (1) assumptions that individuals with ASD are asexual or permanently dependent, which lead caregivers and educators to avoid sexuality topics [4]; (2) curricula that overemphasize risk avoidance rather than fostering autonomy, intimacy, and healthy relationships [5]; (3) a lack of instructional approaches adapted to ASD-specific cognitive and behavioral characteristics, such as a preference for concrete, visual, and highly structured learning, and challenges with interpreting social cues and implicit norms [6]; and (4) limited access to peer-learning environments where informal sexuality knowledge is typically developed [7]. As a result, adolescents with ASD experience greater exposure to harm, including a 2 to 3 times higher risk of sexual victimization (30%‐50% lifetime prevalence) compared with neurotypical peers [8,9], alongside difficulties recognizing consent and personal boundaries [10]. Despite these risks, adolescents with ASD often display developmentally typical sexual curiosity, which is frequently misunderstood as problematic behavior, further impeding their access to affirmative and accurate sexual health education [11].

Traditional school-based programs generally rely on abstract verbal instructions, implicit social rules, and group discussions [9], pedagogical methods that do not adequately address the processing preferences and social learning challenges characteristic of ASD. Although some adapted interventions using visual supports, role-play, behavioral modeling, and caregiver participation have shown potential benefits [10,12], they remain resource intensive, highly dependent on trained specialists, and often limited to high-income settings, thus restricting equitable scale-up [11,12].

Digital health technologies provide an opportunity to address these barriers specifically within the context of sexual health education. Mobile apps, interactive videos, and virtual learning environments can deliver content visually, sequentially, and at a self-paced rhythm aligned with ASD learning patterns [13]. Privacy features allow adolescents to engage with sensitive topics without fear of stigma or embarrassment [14], which is particularly important for those who struggle with discussing sexuality face-to-face. Additionally, cultural and linguistic adaptation can be built directly into platforms, supporting contextual relevance in settings where sexual health remains taboo or tightly regulated.

Despite these benefits, only a small number of digital interventions have focused specifically on sexual health for autistic adolescents. Reviews of technology-based learning for neurodiverse youth [13] and reviews of sex education for people with disabilities more broadly [14] have not examined the unique intersection of ASD-specific pedagogy, adolescent development, sexual content, and digital delivery. Moreover, implementation in low- and middle-income countries (LMICs) is largely absent. Limited digital infrastructure, lower availability of trained providers, and cultural discomfort surrounding discussions of sexuality create additional barriers to uptake and sustainability.

Given these gaps, this scoping review maps digital sexual health interventions for adolescents with ASD, describing digital modalities, educational foundations, implementation contexts, and reported outcomes. We also identify 3 cross-cutting gaps (ie, limited participatory co-design, underrepresentation of girls, and scarce implementation in LMIC settings), which are analyzed in the Discussion to inform future directions.

## Methods

### Study Design

This review follows the scoping methodology originally outlined by Arksey and O’Malley and later refined by Levac et al [15], incorporating the latest methodological recommendations from the Joanna Briggs Institute [16]. The reporting process adheres to the PRISMA-ScR (Preferred Reporting Items for Systematic Reviews and Meta-Analyses extension for Scoping Reviews) checklist [17].

### Review Questions

The review aims to address the following questions:

1. What types of digital health interventions have been developed to deliver sexual health education to adolescents with ASD?
2. How are these interventions designed and implemented in practice?
3. What outcomes, such as educational, behavioral, or usability-related, have been reported?
4. What evidence gaps and contextual challenges emerge from the current literature?

### Eligibility Criteria

The inclusion and exclusion criteria were developed based on the PCC (population, concept, context) framework recommended by the Joanna Briggs Institute Manual for Evidence Synthesis (Table 1).

**Table 1.** Inclusion and exclusion criteria.

PCC component	Inclusion criteria	Exclusion criteria
Population	Adolescents aged approximately 10‐19 y diagnosed with ASD^a^; OR studies involving young adults, caregivers, or practitioners if findings are directly relevant to adolescent sexual health education in ASD	Studies involving only adults with ASD where findings lack relevance to adolescents Studies focused solely on non-ASD neurodevelopmental disorders
Concept	Sexual health education delivered in a digital format, including mobile apps, web platforms, video modeling, virtual simulations, digital social stories, or tele-education Interventions focused on sexual knowledge, boundaries, consent, relationships, hygiene, or online safety	Interventions unrelated to sexual health content Tools designed exclusively for skill areas not linked to sexuality (eg, employment and general social skills)
Context	Any educational, clinical, family, or community setting All cultural and socioeconomic contexts	Studies focused solely on institutional care settings without educational intent
Study design	All empirical study types (RCTs^b^, pilots, mixed methods, qualitative) and co-design or framework studies relevant to digital tools Peer-reviewed journal articles	Reviews, editorials, and commentaries without original data Conference abstracts without full text
Language	Published in English	Non-English publications
Publication date	2010‐2025	—^c^

a ASD: autism spectrum disorder.

b RCT: randomized controlled trial.

c Not applicable.

Studies published from January 2010 to June 2025 were included because this period reflects major advancements in digital health technologies and their integration into health education, including sexuality education for neurodiverse populations. Earlier digital interventions lacked comparable accessibility and interactive features, making them less relevant to current implementation contexts. Only English-language publications were included due to resource and feasibility constraints in translating non-English texts. While this may introduce language bias, limiting the review to English publications ensured consistency in screening and methodological appraisal, although some evidence from non-English-speaking regions may be underrepresented.

### Search Strategy

A comprehensive search was conducted across six major electronic databases: PubMed/MEDLINE, Scopus, CINAHL, ERIC, PsycINFO, and Web of Science. The search strategy combined controlled vocabulary (eg, Medical Subject Headings [MeSH] terms) with relevant free-text keywords related to ASD, adolescence, sexual health education, and digital health interventions. An example of the search string used in PubMed was as follows: (“Autism Spectrum Disorder”[MeSH] OR autism OR ASD) AND (“Adolescents”[MeSH] OR adolescent OR youth) AND (“Sex Education”[MeSH] OR sexual health) AND (“Digital Health”[MeSH] OR eHealth OR mHealth OR digital intervention). To enhance the comprehensiveness of the review, the reference lists of all included studies were manually screened for additional eligible articles that were not captured through database searches.

### Study Selection and Screening Process

All retrieved citations were imported into EndNote for systematic deduplication. The remaining records were then uploaded into Rayyan, a web-based screening platform, to facilitate an organized and blinded review process. Before formal screening, both reviewers conducted a pilot calibration exercise using 20 randomly selected citations from the initial search results (n=243). This was done to refine operational definitions and ensure a shared understanding of the eligibility criteria. Once sufficient agreement was achieved, independent title or abstract screening proceeded for all remaining records. Following calibration, 2 independent reviewers screened titles and abstracts using predefined inclusion and exclusion criteria. Full texts were retrieved for all articles deemed potentially relevant. Discrepancies at any stage were discussed, and if consensus could not be reached, a third reviewer served as adjudicator. This multistep process strengthened the reliability and reproducibility of study selection.

### Data Extraction

A structured and pilot-tested data extraction form was used to ensure consistent and comprehensive data collection across all eligible studies. Extracted information included authorship, publication year, country of origin, study design, participant characteristics, digital modality and duration, theoretical or pedagogical frameworks, educational content and delivery strategies, outcome domains, key findings, and reported limitations or implementation barriers. Data extraction was performed independently by 2 reviewers. Any discrepancies in extracted information were resolved through discussion and consensus; when agreement could not be reached, a third reviewer was consulted to adjudicate. This process ensured accuracy and minimized individual reviewer bias throughout the synthesis.

### Quality Appraisal

Although not mandatory in scoping reviews, a methodological quality assessment was conducted to enhance the interpretative rigor of the findings. The Mixed Methods Appraisal Tool (2018) was used to evaluate each study across 5 design-specific criteria. Two reviewers independently completed the appraisal, resolving any disagreements through discussion and third-reviewer adjudication when necessary. The appraisal was used to contextualize findings rather than to exclude studies.

Overall, the quality of included studies was moderate. Approximately one-quarter of the studies demonstrated high methodological quality (scores of 4/5), typically characterized by clearly defined outcomes and appropriate study designs, including randomized trials and multisite implementations. The majority of studies scored 3 out of 5, reflecting acceptable methodological rigor but common limitations, such as small sample sizes, lack of control groups, and short intervention durations. Lower-quality studies (scores of ≤2/5), primarily narrative or commentary pieces, provided useful context but limited empirical evidence. A full scoring results are presented in the *Results* section (Table 2).

**Table 2.** Quality of study assessment.

Authors, country	MMAT^a^ score (out of 5)	Comments
Chan and John [18], USA	1	Not an empirical study; and lacks data collection, analysis, or measurable outcomes.
Strauss [19], the Netherlands	4	Strong RCT^b^ design, clear measures, and lacks digital delivery and generalizability.
Lou and Arriaga [20], USA	3	Exploratory, appropriate design, and lacks outcome depth and scope.
Teti et al [21], USA	3	Qualitative depth, small sample, and no intervention tested.
Drozdowicz et al [22], USA	4	Well-executed implementation, and lacks ASD^c^-specific data and long-term outcomes.
Pugliese et al [23], USA	4	Feasibility well assessed and pilot scope limits generalization.
Zervogianni et al [24], multicountry	3	Framework development, strong stakeholder input, and lacks effectiveness testing.
Gil-Llario et al [3], Spain	4	Pre-post design, positive results, and small sample and no long-term follow-up.
Chin and Ramachandram [10], Hong Kong	3	Clear pre-post design and lacks control group and large sample.
Ballan and Freyer [11], USA	1	Expert opinion and lacks empirical data.
Shakuri and Alzahrani [25], Saudi Arabia	3	Clear qualitative insights and no intervention tested.
Camilleri et al [26], UK	3	Exploratory design, good engagement data, and lacks outcome rigor.
Panagiotakopoulou et al [27], Greece	3	Focused parent perspective and lacks youth input and intervention.
Quayle et al [28], UK	2	Relevant insights, not ASD specific, and lacks outcome data.
Oğur and Olçay [29], Turkey	3	Clear intervention protocol, small sample, and adult focused.
Wang et al [30], China	3	Important gender-specific insights and lacks adolescent voices and tool testing.

a MMAT: Mixed Methods Appraisal Tool.

b RCT: randomized controlled trial.

c ASD: autism spectrum disorder.

### Data Analysis and Synthesis

Extracted data were organized into structured summary tables capturing key study features, intervention characteristics, and reported outcomes. A narrative synthesis was then conducted using a combined inductive-deductive thematic approach. Deductive coding was informed by the review objectives and a priori categories, such as digital modality, educational strategies, implementation context, and outcome domains. In parallel, inductive coding allowed new concepts to emerge directly from the data, including participant engagement needs, caregiver involvement, and cultural adaptation factors. Themes were iteratively refined through reviewer discussion to ensure consistency and accuracy. Studies were grouped by platform type (eg, mobile apps, web-based interventions, and interactive video tools) and interpreted alongside their methodological quality to contextualize confidence in the findings. Recurring evidence gaps, such as limited sample diversity, scarce LMIC representation, and a lack of standardized behavioral outcomes or long-term follow-up, were also identified to inform directions for future digital sexual health intervention development.

## Results

### Searching Result

**Figure 1.**
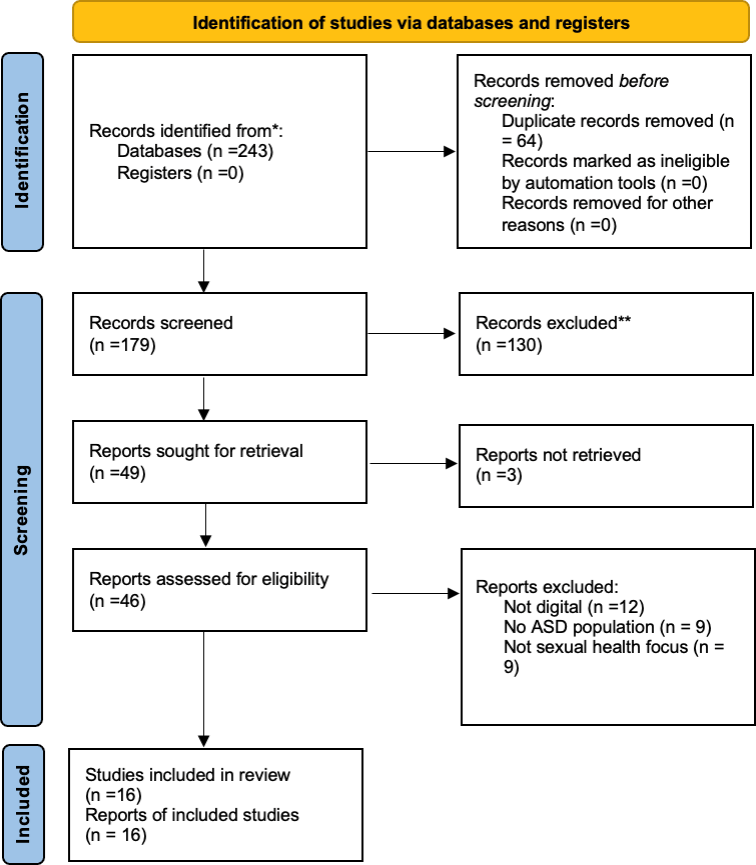
PRISMA (Preferred Reporting Items for Systematic Reviews and Meta-Analyses) 2020 flow diagram for new scoping reviews, which included searches of databases and registers only. ASD: autism spectrum disorder.

The PRISMA-ScR flow diagram presents the full screening process. A total of 243 records were identified across databases, with 64 duplicates removed. Following title and abstract screening, 130 records were excluded, leaving 49 full-text articles assessed for eligibility. Three articles could not be retrieved despite attempts to contact authors. Ultimately, 16 studies met the inclusion criteria and were included in this scoping review (Figure 1).

### Study Characteristics

All 16 included studies were published between 2012 and 2025. Study designs were diverse, including randomized controlled trials, mixed-methods feasibility studies, qualitative exploratory research, participatory framework development, and case series. Research was conducted in the United States, the United Kingdom, Spain, Turkey, China, Greece, Saudi Arabia, Hong Kong, and multicountry collaborations. Notably, 8 studies directly implemented digital sexual health interventions or closely related digital training, whereas 8 studies explored contextual needs, stakeholder perspectives, theoretical frameworks, or digital adoption challenges without delivering a formal intervention. Studies including broader age groups (eg, young adults) were retained when their findings were directly relevant to adolescent-focused intervention design. Participant groups included autistic adolescents, autistic young adults, parents of autistic youth, clinicians, and digital health practitioners. Reporting of demographic and diagnostic characteristics was highly variable, as summarized in Table 3.

**Table 3.** Characteristics of included studies and summary of findings.

Authors, country	Study design and methodology	Population characteristics	Digital intervention details	Educational content and delivery	Outcomes measured	Key findings and limitations
Chan and John [18], USA	Review with clinical guidance	*No sample and narrative guidance only*	None	Guidance for nurse practitioners on how to discuss sexuality with ASD^a^ youth; not a formal intervention	None	Emphasizes early, tailored, and clear communication about sexual health with ASD youth. Not a digital intervention; not a research study
Strauss et al [19], USA	RCT^b^; quantitative, pre-post with control group	Adolescents with ASD, n=96, age 12‐18 y, mixed gender	Face-to-face psychosexual training (tackling teenage program)	Manualized group sessions on puberty, boundaries, consent, and relationships using visual aids	Knowledge, self-esteem, social functioning, ASD-specific sexual behaviors	Significant improvement; limited by lack of digital tools and generalizability
Lou and Arragia [20], USA	Prototype development and exploratory pilot	Adolescents and young adults with ASD, n=14, age 16‐21 y	eLIFE social network application	Social support and communication via mobile app	User engagement and perceived usefulness	Feasibility shown, limited sample, and not sexual health specific
Teti et al [21], USA	Qualitative comparative study	Youth with ASD and caregivers, n=10 dyads, youth age 16‐21 y	No specific tool tested	Need for structured and multimedia sexual education	Themes on miscommunication, autonomy, and relationships	Support for structured education, small sample, and no intervention
Drozdowicz [22], USA	Multisite implementation study using synchronized videoconferencing with standardized patients	Psychiatry trainees and clinical teams, n=42 (not ASD specific)	Educational videoconference sessions involving standardized patient scenarios and delivered synchronously across sites	Sexual health communication training using video-based standardized patient interactions and real-time delivery with feedback	Participant engagement, knowledge gains, comfort in addressing sexual health	Improved comfort and preparedness reported; not ASD specific; no long-term follow-up
Pugliese [23], USA	Pilot feasibility study; mixed methods	Youth with ASD, n=22, age 10‐17 y; parents involved	Parent-mediated digital materials	Anatomy, consent, communication, and parent led	Knowledge gain and parent-teen interaction	Feasible and acceptable and small pilot scope
Zervogianni et al [24], multicountry	Participatory framework development	Individuals with ASD, n=31, mixed age (including adolescents)	Multiple platforms considered	Co-created framework for digital interventions	Process evaluation and stakeholder satisfaction	Robust framework and no implementation or effectiveness measured
Gil-Llario et al [3], Spain	Quantitative and pre-post design	Youth with ASD, n=27, age 12‐18 y	Video-based and web-delivered	Anatomy, consent, and boundaries; and interactive videos	Knowledge and acceptability	Improved knowledge, small sample, and no follow-up
Siah [10], Hong Kong	Pilot study and single group pre-post	Youth with ASD, n=18, age 13‐17 y	Video modeling (3 sessions)	Sexual boundaries, hygiene, and behaviors	Knowledge gain and behavioral understanding	Increased knowledge, no control group, and small sample
Ballan and Freyer [11], USA	Narrative or expert commentary	*No sample; narrative review*	None	Critique of existing nondigital approaches	None	Calls for inclusive digital tools; not empirical
Shakuri and Alzahrani [25], Saudi Arabia	Qualitative study using semistructured interviews with parents	Parents only, n=15, adolescents represented indirectly	No direct intervention and explored digital needs and barriers	Explored parental perceptions of content needs and discussed digital adaptation opportunities	Themes related to cultural, religious, and informational barriers	Strong parental demand for culturally sensitive, digital sexual health resources, and lacks intervention testing
Camilleri [26], UK	Exploratory qualitative study	Autistic youth and parents, n=12, youth age 10‐18 y	Digital social stories and co-designed	Tailored social-sexual themes	Engagement and parent satisfaction	Improved communication and limited by exploratory scope
Panagiotakopoulou et al [27], Greece	Qualitative and interviews with parents	Parents of adolescents with ASD, n=14, adolescents indirectly described	No direct intervention	Need for structured and digital tools	Parental perceptions and barriers	Preference for mobile, visual tools, and no youth data
Quayle [28], UK	Qualitative study involving semistructured interviews with child protection and mental health practitioners	Practitioners only, n=20, *not ASD specific*	Discussion of digital health tools (apps, online therapy, AI^c^ screening) used or envisioned in abuse prevention services	Focused on service-level integration and digital design for sensitive topics	Perceptions of feasibility, ethical concerns, and implementation barriers	Digital tools viewed as promising but complex to implement and lacking empirical outcome data and ASD focus
Oğur and Olçay [29], Turkey	Case series	Adults with ASD, n=3, age 21‐23 y, all men	Video BST^d^ and digital scenes	Prevention of online sexual abuse	Skill acquisition, competency	Effective training; limited adolescent generalizability
Wang [30], China	Qualitative and interviews with parents	Parents of autistic girls, n=11, age 10‐17 y	No formal tool	Parental needs for digital sexual education	Unmet needs and preferred formats	Call for visual, gender-sensitive tools and lacks youth voice

a ASD: autism spectrum disorder.

b RCT: randomized controlled trial.

c AI: artificial intelligence.

d BST: behavioral skills training.

### Digital Intervention Modalities and Implementation

Among the 16 included studies, 8 evaluated digital tools. These interventions primarily used, including interactive video-based sexual education (eg, anatomy, boundaries, and consent), video modeling, and behavioral skills training for online exploitation prevention, mobile and web-based platforms, including social stories and facilitated parental modules, and prototype digital applications supporting psychosexual communication. The remaining 8 studies did not implement a digital intervention but provided essential contextual evidence about acceptability, cultural relevance, and caregiver and professional perspectives that inform digital tool development.

Video-based education was the most frequently used modality. For instance, Gil-Llario et al [3] and Siah et al [10] implemented interactive and modeling-based digital videos to deliver content on consent, boundaries, and appropriate sexual behavior. Similarly, Çimen Oğur and Olçay [29] used video visual scene displays in behavioral skills training to teach online sexual abuse prevention. Pugliese et al [23] and Camilleri et al [26] incorporated parent-mediated modules and co-designed digital social stories, respectively, emphasizing personalized learning.

Mobile and web-based technologies were also explored. Lou and Arriaga [20] tested eLIFE, a prototype social network app aimed at enhancing social communication among youth with ASD, and Zervogianni et al [24] proposed a multiplatform, evidence-based co-design framework for digital tool development. While not always focused specifically on sexual health, these studies revealed key design principles applicable to future interventions. In contrast, several studies [11,27,30] emphasized the need for accessible, culturally responsive, and multimedia sexual health education tools but reported no direct implementation. Parents, in particular, expressed strong preferences for mobile-friendly, visually rich, and repeatable learning formats tailored to the cognitive and developmental profiles of their children.

### Educational Content and Delivery Strategies

The digital interventions identified varied in their educational content and delivery strategies. Commonly addressed topics included sexual anatomy, personal hygiene, consent, social boundaries, healthy relationships, and online safety. Interactive video formats and story-based learning were used to model both appropriate and inappropriate behaviors, particularly in studies with youth aged 13 to 18 years. Parent-mediated tools [23] often used structured modules with guided discussions, while other programs [26] emphasized autonomy and co-creation with autistic youth. Despite this diversity, few studies reported the use of established theoretical frameworks, and most lacked standardized instructional approaches, limiting cross-comparability. Duration and frequency of interventions also varied, with only 3 studies clearly reporting session length and instructional pacing.

### Outcome Domains and Reported Effects

The most frequently measured outcomes were sexual health knowledge, behavioral understanding, skill acquisition, and acceptability or usability. Quantitative studies such as those by Gil-Llario et al [3] and Siah et al [10] reported significant gains in sexual knowledge postintervention. In Çimen Oğur and Olçay [29], participants demonstrated improved behavioral skills for resisting online exploitation. Pugliese et al [23] reported enhanced parent-child communication, while Camilleri et al [26] noted increased engagement and satisfaction among both youth and parents.

Qualitative studies underscored the emotional, relational, and cognitive barriers that youth with ASD face regarding sexuality. Teti et al [21] and Wang et al [30] highlighted themes of communication breakdown, information gaps, and parental uncertainty, while Lou and Arriaga [20] emphasized the socioemotional impact of peer-based digital tools. However, many of these studies did not measure outcomes longitudinally, limiting insight into sustained effectiveness.

### Methodological Limitations and Evidence Gaps

Across studies, common limitations included small samples, absence of control groups or randomization, nonstandardized outcomes, and short follow-up periods. Three recurrent evidence gaps were reported: (1) limited participatory co-design with autistic adolescents, (2) underrepresentation of female participants and lack of gender-specific content, and (3) few implementations in LMICs. These gaps were noted but not tested as effect modifiers. Only Wang et al [30] addressed female-specific educational gaps, emphasizing the lack of tailored content. Co-design with autistic adolescents was inconsistently applied, although Zervogianni et al [24] and Camilleri et al [26] provided important examples of participatory development.

### Thematic Patterns

The narrative synthesis revealed several recurring patterns across the included studies. One prominent theme was the need for personalization and visual interactivity within digital sexual health interventions designed for adolescents with ASD. Interventions that used video modeling, interactive storytelling, or visual scene displays were notably more effective in enhancing comprehension and engagement, particularly among youth with ASD, who often benefit from visual structure and concrete representations of social behavior [10,26,29].

Another consistent finding was the strong demand from caregivers for mobile-accessible, culturally sensitive, and repeatable educational content. Parents expressed a clear preference for digital tools that could be used flexibly within the home, reflect local sociocultural values, and offer structured, developmentally appropriate guidance on sexual health topics [23,27,30].

A third thematic pattern highlighted critical gaps in the implementation and evaluation of digital interventions. Many studies lacked contextual diversity, with limited representation of LMICs, and often failed to incorporate adolescent end users in the design process [11,24]. Inadequate attention to co-design and cultural adaptability may reduce the acceptability and effectiveness of digital interventions across different settings. Moreover, few studies included long-term follow-up or robust behavioral outcome measures, limiting the ability to assess sustained impact [3,20].

### Methodological Quality Appraisal

The overall methodological quality of the included studies varied. High-quality studies, such as those by Strauss et al [19], Drozdowicz et al [22], Pugliese et al [23], and Gil-Llario et al [3], scored 4/5 on Mixed Methods Appraisal Tool, reflecting strong designs with clear outcomes, although some lacked digital focus or generalizability. Moderate-quality studies (3/5), including Lou and Arriaga [20], Teti et al [21], and Siah et al [10], showed feasibility or insight but were limited by sample size or lack of control groups. Lower scores (≤2/5), such as Chan and John [18] and Ballan and Freyer [11], were nonempirical or lacked data collection, limiting their evidentiary value (Table 2).

## Discussion

### Principal Findings

This scoping review maps the emerging landscape of digital sexual health education for adolescents with ASD. Across diverse study designs and settings, the evidence indicates early effectiveness for formats that align with ASD learning characteristics, particularly visually rich, structured, and repeatable content, while also revealing systemic gaps that limit generalizability and equity. Below, we interpret key patterns and translate them into actionable implications for design, implementation, and evaluation.

Expanding upon foundational reviews by Pownall et al [31] and Dewinter et al [32], which underscored the scarcity of structured, inclusive sex education for neurodiverse individuals, this review uniquely updates the evidence base with a focus on digital modalities. Previous syntheses primarily emphasized content deficits and the inadequacy of traditional education formats for autistic learners. In contrast, our findings highlight a tangible shift toward digital, video-based, and mobile platforms that are more aligned with the cognitive, sensory, and social processing styles of autistic adolescents. This shift marks a substantial evolution in approach, emphasizing not only what is taught, but how and by whom it is delivered. Unlike earlier reviews that often conflated multiple developmental disabilities, this scoping review centers specifically on ASD, revealing critical insights into learning preferences, gender disparities, and digital engagement unique to this population. A notable distinction is the emerging but still limited incorporation of co-production and participatory design, reflecting a movement toward user-centered innovation in health education. This is particularly significant, given the evidence that autistic individuals benefit from interventions designed with their direct input [24].

A central theme across the reviewed literature is the demonstrated effectiveness of video-based interventions in enhancing sexual knowledge and social comprehension. Studies by Gil-Llario et al [3], Siah et al [10], and Çimen Oğur and Olçay [29] illustrate that interactive video, especially those incorporating role modeling, repetition, and guided narration, can support autistic adolescents in understanding abstract concepts, such as consent, bodily autonomy, and relationship boundaries. These pedagogical strategies are fundamentally aligned with the cognitive characteristics of ASD [26], including a preference for visual, concrete, structured learning, reduced social inference skills, and challenges in generalization across contexts. Therefore, future digital interventions should increasingly incorporate visual prompts, stepwise sequencing, clearer discrimination between appropriate and inappropriate behaviors, and opportunities for individualized feedback and repeated practice.

Another critical finding emerging from the reviewed studies is the central role of caregivers in facilitating sexual health learning among autistic adolescents. Evidence from Pugliese et al [23] and Panagiotakopoulou et al [27] shows that parents overwhelmingly favor visually rich, repeatable, and culturally adaptable content—particularly in contexts where formal sex education is limited or inconsistently delivered to neurodiverse youth. These preferences reflect the widespread reliance on the family as a primary learning environment and suggest that digital tools are more likely to be used when they can be integrated into existing home routines and communication patterns.

Despite these promising developments, the body of evidence remains fragmented and unevenly distributed. Most studies originate from high-income settings, with limited consideration for sociocultural values, digital access, and educational policies in LMICs. This geographic concentration reduces the transferability of findings to resource-constrained settings, where formal sexuality education may be stigmatized or absent. The lack of reporting on cultural or religious context in intervention design also limits understanding of how well existing programs align with local expectations regarding sexual behavior and autonomy. Across studies, intervention samples frequently overrepresented male adolescents, meaning that the needs, experiences, and risks faced by autistic girls remain insufficiently represented. Only a small number of studies, such as Wang et al [30], explicitly examined female perspectives in digital development or outcome reporting. This gender imbalance reflects a broader trend in autism research and raises concerns that current interventions may unintentionally reinforce a male-centric understanding of autistic sexuality.

Taken together, these findings indicate that while digital interventions hold considerable promise in tailoring sexual health education to autistic adolescents’ learning profiles, important equity considerations persist. Variability in sampling, design rigor, cultural contextualization, and gender representation highlights the need for continued refinement before such interventions can be confidently translated into routine education and clinical practice. Strengthening methodological quality and ensuring inclusive design processes will be essential to develop interventions that are not only effective but also broadly relevant and accessible.

Importantly, the underrepresentation of female adolescents with ASD has long been recognized as a systemic gap in autism research and persists. Only a handful of studies, such as Wang et al [30], explored the unique needs and experiences of girls, raising concerns about gender equity in intervention design and delivery. Without targeted inclusion, current tools risk reinforcing a male-centric view of autism that excludes a significant portion of the population. Geographic and socioeconomic disparities also limit the generalizability of current findings. Very few studies examined implementation in LMICs, where digital infrastructure, cultural norms, and access to health education differ markedly from high-income contexts. As Ballan and Freyer [11] argue, failing to address these structural factors risks exacerbating existing inequities, particularly for youth in marginalized communities. Future tools must therefore ensure gender-responsive adaptation of content, particularly around puberty, menstrual health, privacy, and romantic relationships.

Collectively, these findings affirm that digital interventions hold transformative potential for delivering inclusive, accessible, and developmentally appropriate sexual health education to adolescents with ASD. However, a shift from proof-of-concept studies to large-scale, context-sensitive, equity-driven research is urgently required. This includes participatory design methods that meaningfully involve autistic adolescents, families, and educators in LMICs to ensure interventions are relevant, feasible, and ethically grounded.

### Limitations

This scoping review has several limitations that should be considered when interpreting the findings. First, significant methodological heterogeneity across the included studies, particularly in sample sizes, designs, and outcomes, restricted the ability to compare results directly or assess effectiveness at scale. Second, limiting the search to English-language publications may have led to the exclusion of relevant evidence from non-English-speaking regions, potentially underrepresenting interventions developed in culturally diverse contexts. Third, most studies were conducted in high-income countries, which limits the generalizability of findings to low- and middle-income settings where resources, digital access, and sociocultural norms related to sexuality education differ markedly. Fourth, although a methodological quality appraisal was conducted, many studies provided only preliminary or feasibility data, making it difficult to draw strong conclusions about long-term behavioral impact. Finally, rapid technological evolution means that some digital tools reviewed may already be outdated, affecting the current applicability of results. Collectively, these limitations emphasize the need for more rigorous, inclusive, and globally representative evaluations to strengthen the evidence base for digital sexual health education among autistic adolescents.

### Implications for Nursing Practice

This review underscores the essential role of nurses in facilitating and advocating for digital sexual health education for autistic adolescents. To ensure interventions are effective and sensitive to ASD-specific learning needs, nurses require competencies in both sexual health communication and digital literacy. As caregivers often serve as primary educators on sexuality, nursing support should extend to families by offering structured guidance and training in the confident and appropriate use of digital tools. In addition to direct education, nurses act as key advocates in shaping school and community policies that promote equitable digital access, safeguard privacy, and ensure culturally and linguistically responsive resources, particularly in LMICs where stigma and limited services remain major barriers to sexual health education.

Future research and program development should prioritize participatory co-design involving autistic adolescents, ensure more gender-responsive content that better represents girls, and adopt standardized outcomes with longitudinal or controlled designs to improve evidence strength and real-world applicability. Emerging approaches such as gamification and artificial intelligence–driven personalization hold promise for enhancing engagement and adaptive learning. Through continued leadership in policy, practice, and interdisciplinary collaboration, nurses will play a critical role in ensuring digital interventions are ethical, accessible, and inclusive, ultimately empowering autistic adolescents to build autonomy, agency, and personal safety in their relationships and sexual health.

### Conclusions

Digital health interventions show considerable promise in closing the longstanding gap in sexual health education for adolescents with ASD, particularly when designed to reflect their visual, structured, and self-paced learning needs. Current evidence demonstrates early effectiveness but remains limited by small samples, inconsistent measurement, and underrepresentation of girls and youth from low-resource settings. Moving forward, progress depends on participatory co-design with autistic adolescents, gender-responsive content, and culturally adaptable formats suitable for LMICs. Strengthening methodological rigor through standardized outcomes and longer follow-up will be essential to understanding long-term impact. Ultimately, digitally enabled, inclusive sexual health education can equip autistic adolescents with the autonomy, safety skills, and confidence needed to navigate relationships and well-being throughout their lives.

## Supplementary material

10.2196/79009 Checklist 1 PRISMA-ScR Checklist.

## Abbreviations

ASD: autism spectrum disorder
LMIC: low- and middle-income country
MeSH: Medical Subject Headings
PCC: population-concept-context
PRISMA-ScR: Preferred Reporting Items for Systematic Reviews and Meta-Analyses Extension for Scoping Reviews
